# 16S ribosomal RNA sequencing and molecular serotyping of *Avibacterium paragallinarum* isolated from Indian field conditions

**DOI:** 10.14202/vetworld.2017.1004-1007

**Published:** 2017-08-28

**Authors:** Vihang Vithalrao Patil, Debendranath Mishra, Dilip Vithalrao Mane

**Affiliations:** 1 Department of Biotechnology, Biotechnology Research Centre, College of Computer Sciences and IT, Latur - 413 512, Maharashtra, India; 2 Swami Ramanand Teerth Marathwada University Sub-Centre, Latur - 413 531, Maharashtra, India; 3 Indovax Private Limited, Gurgaon - 122 001, Haryana, India

**Keywords:** *Avibacterium paragallinarum*, HPG-2 PCR, infectious coryza, multiplex polymerase chain reaction, India, 16S ribosomal RNA sequencing

## Abstract

**Aim:**

This study was aimed at identifying Indian field isolates of *Avibacterium paragallinarum* on both molecular as well as serological levels that cause infectious coryza in chickens.

**Materials and Methods:**

Species-specific polymerase chain reaction (HPG-2 PCR), and 16S ribosomal RNA (rRNA) sequencing were employed for molecular identification. Whereas, multiplex PCR technique was used for serological identification of Indian field isolates of *A. paragallinarum*.

**Results:**

All three field isolates were identified as *A. paragallinarum* using HPG-2 PCR. The species-specific PCR results were validated using 16S rRNA sequencing. The partial 16S rRNA sequences obtained from all three isolates showed 96-99% homology with the NCBI database reference strains of *A. paragallinarum*. The aligned partial sequences of 16S rRNA were submitted to GenBank, and accession numbers were obtained. Multiplex PCR-based molecular serotyping showed that there are three serotypes of field isolates of *A. paragallinarum*, namely, strain IND101 is serovar A, strain IND102 is serovar B, and strain IND103 is serovar C.

**Conclusion:**

HPG-2 PCR, 16S rRNA sequencing, and multiplex PCR are proved to be more accurate, sensitive, and reliable diagnostic tools for molecular and serological identification of *A. paragallinarum* field isolates. These diagnostic methods can substitute conventional cultural characterization and would be much valuable to formulate quick and correct prevention and control measures against this detrimental poultry pathogen.

## Introduction

Avian infectious coryza is an acute upper respiratory disease in chickens manifested by common symptoms such as nasal discharge, sneezing, facial edema, and conjunctivitis [1]. It is a cosmopolitan disease observed wherever extensive poultry operations exist. The disease increases cull formation in broilers and reduce egg production in layers substantially (10-40%) [2]. It indicates bacterial disease control is an important issue in poultry farms [3,4]. Infectious coryza is caused by Gram-negative short rod bacterium *Avibacterium paragallinarum*. The bacterium is a slow-growing and fastidious organism needs NAD (factor V) for *in vitro* growth [5]. *A. paragallinarum* is classified into three serotypes, i.e., serovars A, B, and C [6].

Commonly, *A. paragallinarum* gets masked and overgrown by other hemophilic organisms belongs to family *Pasteurellaceae* such as *Avibacterium avium*, *Avibacterium gallinarum*, and *Avibacterium volantium* [7]. Conventionally, the disease is diagnosed merely by symptoms and confirmed by isolating the organism in culture with satellite growth [8].

There is a possibility that regular diagnostic methods are not effective and can misinterpret infectious coryza with other poultry diseases. To overcome this situation, there is a need to use more sensitive and accurate method for diagnosis and confirmation of *A. paragallinarum*. At present, many alternative approaches such as species-specific polymerase chain reaction (HPG-2 PCR) [9], DNA Restriction endonuclease analysis [10], ribotyping [11], ERIC-PCR [12], real-time PCR [13], and 16S ribosomal RNA (rRNA) sequencing [7,14] are employed and found to be more precise and sensitive identifying tools for *A. paragallinarum*. The serovar level recognition of *A. paragallinarum* is widely carried out using HA-HI test [6]. Recently, the multiplex PCR-based molecular serotyping is also getting popular for molecular serotyping of causative agent [15].

In India, infectious coryza was first reported in the 1950s. Since then, it is one of the commonly found diseases in many poultry pockets of the country [16-19]. Unfortunately, the information about outbreaks and disease prevalence is very scanty due to tedious isolation procedure and fastidious nature of this organism [20]. However, modern diagnostic approaches are observed to be more successful in the authentic identification and characterization of *A. paragallinarum* at the molecular level to control disease. To control bacterial diseases, different strategies can be implemented in poultry farms [21-23].

The present study was conducted on species-specific PCR and 16S rRNA sequencing for identification of *A. paragallinarum* field isolates. Multiplex PCR-based molecular serotyping was also performed for very first time in India to sort *A. paragallinarum* field isolates on serological level. The identified three isolates used in this study were previously isolated and studied for their virulence pattern by the present authors from different geographical locations of India between the years 2012 and 2015 [24,25].

## Materials and Methods

### Ethical approval

No ethical approval was necessary to conduct this study.

### Isolates

Three chosen Indian field isolates of *A. paragallinarum* were characterized by authors in the course of 2012-2015 [24,25]. The isolates were revived from storage and used for molecular characterization.

### DNA isolation

Brain-heart infusion broth supplemented with 1% (W/V) sodium chloride, 0.0025% (W/V) reduced nicotinamide adenine dinucleotide, and 1% (V/V) filter-sterilized chicken serum were inoculated with single colony of each isolate and kept for overnight incubation at 37°C. The DNA was extracted from overnight grown bacterial cultures using DNeasy Blood and Tissue DNA Kit (Qiagen, Germany).

### HPG-2 PCR confirmation

The retrieved cultures were reconfirmed for *A. paragallinarum* by performing species-specific PCR (HPG2-PCR). A set of primers F1 (TGAGGGTAGTCTTGCACGCGAAT) and R1 (CAAGGTATCGATCGTCTCTCTACT) were used to amplify species-specific 500 bp fragment of *A. paragallinarum* [9].

### Amplification and sequencing of 16S rRNA

The genomic DNA extracted in the previous steps was used as template DNA. Bacterial 16S region gene was amplified using standard PCR reaction. The primer pair 27F (AGAGTTTGATCMTGGCTCAG) and 1492R (TACGGYTACCTTGTTACGACTT) was used in a PCR reaction with an annealing temperature of 57°C. After amplification, products were purified using Invitrogen PCR product purification kit (Life Technologies, USA) and were directly sequenced using an ABI PRISM BigDye Terminator V3.1 kit (Applied Biosystems, USA). The sequences were analyzed and edited using Sequencing Analysis V5.2 Software (Applied Biosystems, USA) and compared with already published sequences in the GenBank, NCBI, with the help of BLAST tool [26]. The sequences were also submitted to GenBank, NCBI, to obtain unique accession number.

### Multiplex PCR

The genomic DNA extracted was further used as PCR template for multiplex PCR method. Briefly, the PCR reactions were performed for a total volume of 25 µl containing 100 ng of template DNA, 1× PCR buffer, 0.2 mM of each dNTP, 0.2 µM Page serovar-specific primers, and 1.25 units of DNA Taq polymerase. The sequences of primers used for amplification of DNA are as follows:

The amplification steps were carried out as per the standard protocol, and PCR products were analyzed using 2% (W/V) agarose gel electrophoresis [15].

**Table:** 

Primer	Sequence (5’-3’)	Amplicon size (kbp)
ABC forward	GGCTCACAGCTTTATGCAACGAA	-
A reverse	CGCGGGATTGTTGATTTTGTT	0.8
B reverse	GGTGAATTTCACCACACCAC	1.1
C reverse	TAATTTTCTTATTCCCAGCATCAATACCAT	1.6

## Results and Discussion

### HPG-2 PCR confirmation

**Figure-1:**
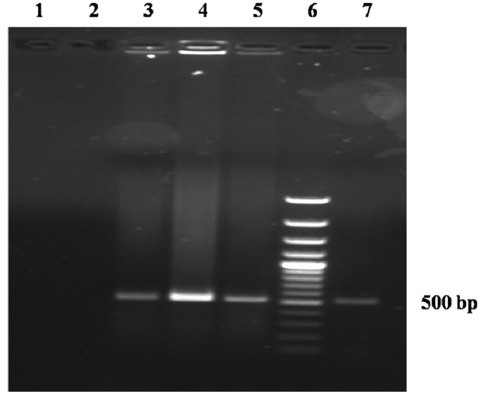
*Haemophilus paragallinarum* 2 polymerase chain reaction confirmation of *Avibacterium paragallinarum* (Lane 1 and 2: No template control, Lane 3, 4, and 5: *A. paragallinarum* isolates, Lane 6: 100 bp plus DNA Ladder, and Lane 7: Reference strain of *A. paragallinarum*).

HPG2-PCR is a sensitive and reliable method for species-specific identification of *A. paragallinarum* [27]. All three isolates after HPG2-PCR produced species-specific 500 bp product (Figure-1). This report is similar to the earlier reports [9,16,27,28].

### Amplification and sequencing of 16S rRNA

The sequences obtained from all three isolates showed 96-99% homology with the NCBI database reference strains of *A. paragallinarum*. The aligned partial sequences of 16S rRNA were submitted to GenBank and accession numbers were obtained (Table 1). These results are in concurrence with the previous findings [14,26].

**Table-1:** NCBI accession number assigned to 16S rRNA sequences of Indian field isolates of *Avibacterium paragallinarum.*

Organism	Strain	Accession number (NCBI)
*Avibacterium paragallinarum*	IND101	KX759522
*Avibacterium paragallinarum*	IND102	KX722532
*Avibacterium paragallinarum*	IND103	KX759523

rRNA=Ribosomal RNA

### Multiplex PCR

In this test, bacterial *HMTp210* gene was amplified using standard PCR reaction. The set of four primers including ABC forward, A reverse, B reverse, and C reverse were used.

**Figure-2:**
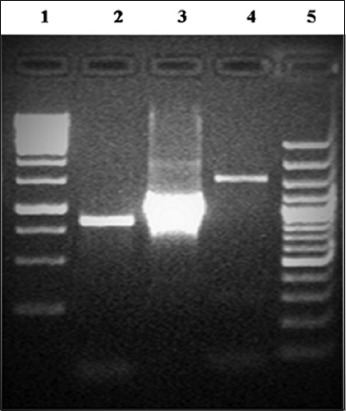
Agarose gel electrophoresis of multiplex polymerase chain reaction products from field isolates of *Avibacterium paragallinarum* (Lane 1 and 5: Molecular weight markers, Lane 2: Strain IND 101, Lane 3: Strain IND 102, and Lane 4: Strain IND 103).

DNA sample of strain IND101 showed amplicon of size 0.8 Kbp which corresponds to detection of serovar A. Further, strain IND102 and strain IND103 generated amplicons of size 1.1 Kbp and 1.6 Kbp, respectively, which matched to serovar B and serovar C (Figure-2 and Table 2). These reports of serovar identification of *A. paragallinarum* are in agreement with other reports [29,30].

**Table-2:** Multiplex PCR assay of *Avibacterium paragallinarum.*

Strain	Amplicon size	Serovar
*Avibacterium paragallinarum* IND 101	0.8 Kbp	A
*Avibacterium paragallinarum* IND 102	1.1 Kbp	B
*Avibacterium paragallinarum* IND 103	1.6 Kbp	C

PCR=Polymerase chain reaction

For preventive measure and a better understanding of a disease, it is essential to identify pathogen on serovar level. Infectious coryza is caused by all three serovars of *A. paragallinarum*, i.e., serovars A, B, and C. In this study, for the very first time, multiplex PCR for serological classification of Indian *A. paragallinarum* was introduced. Through the use of multiplex PCR, this study notably reconfirms the co-occurrence of *A. paragallinarum* serovar B in India [24]. However, earlier report had expressed only *A. paragallinarum* serovar A and serovar C are prevalent in India [19]. Hence, the present study and application of multidimensional modern tools to identify and confirm *A. paragallinarum* serotypes found effective.

## Conclusion

In this study, three field isolates of *A. paragallinarum* were confirmed for their identity using species-specific PCR (HPG-2 PCR) and 16S rRNA sequencing. The isolates were also characterized on the serological level using multiplex PCR method. Both identification tools are found to be more effective and reliable in identification and differentiation of *A. paragallinarum* field isolates from other nonpathogenic avian *Haemophili* and respiratory pathogens. The multiplex PCR analysis of these isolates revealed the co-occurrence of all three serotypes, i.e., A, B, and C of *A. paragallinarum* in India. Application of the present technique found to be simple and sensitive for serological identification of *A. paragallinarum*. Finally, the application of these next-generation diagnostic tools would be much valuable to formulate quick and correct prevention and control measures against this detrimental poultry pathogen.

## Authors’ Contributions

VVP designed and carried out the research work. DM and DVM supervised the research work. VVP drafted the manuscript. DM and DVM revised the manuscript. All authors read and approved the final manuscript.
